# Dysregulated Tfh/B cells and their interactions in neuromyelitis optica spectrum disorder

**DOI:** 10.3389/fimmu.2025.1704282

**Published:** 2026-01-06

**Authors:** Liang Wang, Lei Zhou, Zhouzhou Wang, Jingzi ZhangBao, Wenjuan Huang, Hongmei Tan, Yuxin Fan, Chuanzhen Lu, Jian Yu, Min Wang, Jiahong Lu, Chongbo Zhao, Jun Wang, Chao Quan

**Affiliations:** 1 Department of Neurology and Rare Disease Center, Huashan Hospital, Shanghai Medical College, Fudan University, Shanghai, China; 2 National Center for Neurological Disorders, Shanghai, China; 3 Department of Ophthalmology and Vision Science, Eye and ENT Hospital, Shanghai Medical College, Fudan University, Shanghai, China; 4 Department of Integrative Medicine and Neurobiology, School of Basic Medical Science, Shanghai Medical College, Shanghai Key Laboratory of Acupuncture Mechanism and Acupoint Function, Fudan University, Shanghai, China

**Keywords:** aquaporin 4, B cell, follicular helper T cell, neuromyelitis optica spectrum disorder, pathogenesis

## Abstract

**Background:**

This study aimed to compare the proportions of circulating follicular helper T (Tfh) and B cell subsets, as well as serum levels of cytokines and chemokines, between patients with neuromyelitis optica spectrum disorder (NMOSD) who are anti-aquaporin-4 antibody (AQP4-ab)–positive and healthy controls, and to investigate the interaction mechanisms between Tfh and B cells.

**Methods:**

AQP4-ab–positive NMOSD patients were enrolled during acute attacks and remission phases, along with age- and sex-matched healthy controls. Flow cytometry was used to assess circulating Tfh and B cell subsets. Purified CD19^+^ B cells were cultured alone or co-cultured with CD4^+^CXCR5^+^ Tfh cells for 6 days, with various interventions applied to evaluate alterations in Tfh or B cell phenotypes. Serum and supernatant levels of interleukin (IL)-6, IL-21, CXCL13, and AQP4-ab were measured.

**Results:**

During acute attacks, NMOSD patients exhibited significantly higher proportions of total Tfh, ICOS^+^ Tfh, activated Tfh17, switched memory B cells, double-negative B cells, plasmablasts, and plasma cells, along with elevated serum levels of IL-6, IL-21, and CXCL13. In contrast, the frequencies of activated Tfh1, naive B cells, and transitional regulatory B cell subsets were significantly reduced. Functional assays revealed that Tfh cells promoted B cell proliferation, differentiation, and AQP4-ab production. Conversely, B cell subsets enhanced Tfh cell proliferation, differentiation, and IL-21 secretion; these effects were attenuated by anti-CD20 and anti–interferon-γ (IFN-γ) monoclonal antibodies, but were augmented by anti–IL-10 monoclonal antibody.

**Conclusions:**

Circulating Tfh and B cell subsets are dysregulated in AQP4-ab–positive NMOSD, accompanied by increased levels of IL-6, IL-21, and CXCL13. Reciprocal interactions between Tfh and B cells likely contribute to disease pathogenesis.

## Introduction

Neuromyelitis optica spectrum disorder (NMOSD) is an inflammatory autoimmune disease that predominantly affects the optic nerves and spinal cord, with anti-aquaporin-4 antibody (AQP4-ab) recognized as its most prevalent biomarker (1). During acute attacks, patients typically exhibit elevated Expanded Disability Status Scale (EDSS) scores and require therapeutic interventions such as high-dose methylprednisolone, plasma exchange, or intravenous immunoglobulin. Maintenance therapy in the remission phase commonly includes immunosuppressive agents or biological therapies (2).

Extensive research has been conducted on potential biomarkers associated with AQP4-ab–positive NMOSD, including lymphocyte subsets, cytokines, and chemokines. Of particular significance are T helper 17 (Th17)-related cytokines—such as interleukin (IL)-6, IL-17, and IL-21—which have been implicated in the pathogenesis of inflammatory lesions in NMOSD (3). Additionally, aberrations in follicular helper T (Tfh) cells, B cell subsets, and the chemokine CXCL13 have been documented. Studies indicate that during disease relapse, the frequencies of Tfh cells, switched memory B cells, plasmablasts, and serum levels of CXCL13 are significantly increased compared to those observed during remission and in healthy controls, while the proportions of naive and transitional regulatory B cells are reduced (4–7). Furthermore, reciprocal interactions between T and B cells are increasingly acknowledged as pivotal in the development and progression of NMOSD (8). *In vitro* evidence demonstrates that Tfh cells facilitate B cell proliferation, differentiation, and antibody production through the secretion of IL-21, whereas B cells can reciprocally modulate Tfh cell differentiation (9–12). Nevertheless, the precise dynamics of Tfh and B cell subset interactions in AQP4-ab–positive NMOSD patients remain incompletely characterized.

In this study, we sought to comprehensively characterize changes in circulating Tfh and B cell subsets, as well as serum profiles of cytokines and chemokines, in NMOSD patients during acute attacks and remission phases compared to healthy controls. We further aimed to investigate their correlations with clinical disability scores to identify potential biomarkers and to gain insights into the interplay between circulating Tfh and B cells.

## Materials and methods

### Participants and data collection

We enrolled AQP4-ab–positive NMOSD patients from the Department of Neurology at Huashan Hospital, along with age- and gender-matched healthy controls. All patients had undergone serum AQP4-ab testing using a fixed cell-based indirect immunofluorescence assay (Euroimmun AG, Lüebeck, Germany) as part of routine diagnostic procedures and fulfilled the 2015 international diagnostic criteria for AQP4-ab–positive NMOSD (13). Demographic and clinical characteristics were recorded and are summarized in **Table 1**.

**Table 1:** Demographic and clinical characteristics of AQP4-ab-positive NMOSD patients and control subjects.

Characteristics	Acute attack (N = 21)	Remission (N = 21)	Control group (N = 21)	P value
Female (%)	19 (90.5)	18 (85.7)	17 (81.0)	0.901
Age at recruitment, y	44.2 ± 10.8	46.9 ± 19.2	41.2 ± 10.7	0.451
Antibody titer	1:100 (1:10-1:3200)	1:100 (1:10-1:3200)	—	0.945
EDSS score at recruitment	4 (3-7.5)	2 (0-3)	—	< 0.001
Clinical manifestation (%)	Optic neuritis (66.7), myelitis (33.3)	—	—	—
Immunotherapy (%)	High-dose methylprednisolone (100), intravenous immunoglobulin (14.3)	Azathioprine (28.6), mycophenolate mofetil (71.4)	—	—
Oral predinisone, mg qd	—	5 (0-10)	—	—

Both NMOSD patients experiencing acute attacks and those in remission were included. Acute attacks encompassed either the initial episode or relapse, defined as the emergence of new neurological deficits or worsening of pre-existing symptoms persisting for more than 24 h (14). Patients during acute attacks were recruited prior to initiation of high-dose methylprednisolone. Patients in remission had received at least 6 months of maintenance treatment. Exclusion criteria included seronegativity for AQP4-ab and prior administration of biological therapies.

This study was approved by the Medical Ethics Committee of Huashan Hospital, Fudan University. Written informed consent was obtained from all participants.

### Flow cytometry analysis

Peripheral venous blood samples were collected in EDTA-containing tubes, with 100 µl aliquoted into two separate tubes; serum samples were collected simultaneously collected. Whole blood samples were stained with fluorescently labeled monoclonal antibodies at 4 °C in the dark for 30 min. Following red blood cell lysis using FACS Lysing Solution (BD Biosciences, USA), samples were washed twice and resuspended in 200 µl of phosphate-buffered saline (PBS) supplemented with 0.5% fetal bovine serum (FBS). The frequencies of circulating Tfh and B cell subsets were analyzed using an Attune Acoustic Focusing Cytometer (Thermo Fisher Scientific, USA). Isotype control staining was performed to ensure accurate gating strategies (**Supplementary Tables 1, 2**).

### Flow cytometry sorting and cell culture

Detailed experimental protocols have been described previously (11). Briefly, peripheral blood mononuclear cells (PBMCs) were isolated from 10 ml of heparinized blood via Ficoll-Hypaque density gradient centrifugation (Sigma-Aldrich, USA). PBMCs were washed twice, resuspended, and filtered through a 70 μm strainer (Corning, USA). Cells were then loaded onto a FACSMelody cell sorter (BD Biosciences, USA) for isolation of circulating CD4^+^CXCR5^+^ Tfh cells, CD19^+^ B cells, CD19^+^CD27– naive B cells, and CD19^+^CD27^+^ memory B cells.

Approximately 50,000 purified Tfh cells or B cell subsets were labeled with 1 μM CFSE (Peprotech, USA) and seeded into individual wells to establish monoculture or co-culture systems. Cells were maintained in complete RPMI 1640 medium containing L-glutamine and 25 mM HEPES (Gibco, USA). Each well received 100 ng/ml Staphylococcal enterotoxin B (SEB, Sigma-Aldrich, USA), with final volumes adjusted to 200 μl. Experimental conditions were set up in duplicate. Recombinant human IL-6 (PeproTech, USA) was added at concentrations ranging from 0 to 100 ng/ml. IL-6R–Fc and IL-21R–Fc (R&D Systems, USA) were added at a concentration of 10 μg/ml. In additional intervention experiments, anti-CD20 (Abmole, USA), anti–interferon-γ (IFN-γ) (BioXcell, USA), or anti–IL-10 (eBioscience, USA) monoclonal antibodies were added individually at 10 μg/ml, and IL-12 (10 ng/ml, PeproTech, USA) was added to promote Tfh cell differentiation. Cultures were incubated at 37 °C in a humidified atmosphere with 5% CO_2_ for 6 days. At the end of the culture period, cells and supernatants were collected for subsequent analyses by flow cytometry and enzyme-linked immunosorbent assay (ELISA). Flow cytometry reagent details are provided in **Supplementary Table 2**. For flow analysis, cells were washed, resuspended, and incubated with 0.1 μl of Viability Dye 780 (Peprotech, USA) at 4 °C for 30 min. After washing, cells were incubated with 1 μl of each panel-specific fluorescent monoclonal antibody. For intranuclear BCL6 staining, cells were fixed and permeabilized using the FOXP3/Transcription Factor Staining Buffer Kit (eBioscience, USA).

### Cytokine, chemokine, and antibody detection

Serum and culture supernatant samples were centrifuged immediately after collection and stored at −80 °C until analysis. Levels of IL-6, IL-21, CXCL13, and AQP4-ab levels were quantified using commercially available enzyme-linked immunosorbent assay (ELISA) kits (LMAI Bio, China) according to the manufacturer’s instructions. All assays were performed in duplicate.

### Statistical analysis

Flow cytometry data were analyzed using FlowJo X10.0 software (FlowJo, USA) for gating of FCS files. Statistical analyses were performed using SPSS 22.0 (SPSS Inc., USA), while graphical visualization was conducted using GraphPad Prism 7 software (GraphPad Software Inc., USA) and R (version 4.1.3, http://www.r-project.org/). Quantitative data are presented as mean ± standard deviation (SD) or median and range, depending on the normality of distribution. Comparisons across multiple groups were carried out using one-way ANOVA or Welch’s ANOVA, based on the homogeneity of variances. *Post hoc* pairwise comparisons were performed using Tukey’s test or the Games–Howell test, as appropriate. Categorical data were compared using the chi-square test or Fisher’s exact test. Spearman correlation analysis was employed to assess the association between clinical disability scores and immunological parameters in all AQP4-ab–positive NMOSD patients during both acute attacks and remission phases. Statistical significance was set at P < 0.05.

## Results

### Population characteristics

A total of 63 independent participants were enrolled in this study, comprising 21 NMOSD patients with NMOSD during acute attacks, 21 NMOSD patients in remission, and 21 healthy controls. The mean ages were 44.2, 46.9, and 41.2 years, respectively; the proportions of female participants were 90.5%, 85.7%, and 81.0%, with no statistically significant differences among the three groups (P = 0.451 and P = 0.901, respectively). Expanded Disability Status Scale (EDSS) scores at enrollment were significantly higher in the acute attack group than in the remission group (P < 0.001). Three patients (3/21, 14.3%) had received a single dose of intravenous immunoglobulin prior to sample collection. Among patients in remission, the median daily oral prednisone dosage was 5 mg (range: 0–10 mg).

### The proportions of Tfh and B cell subsets were dysregulated during acute attacks

**Figure 1:**
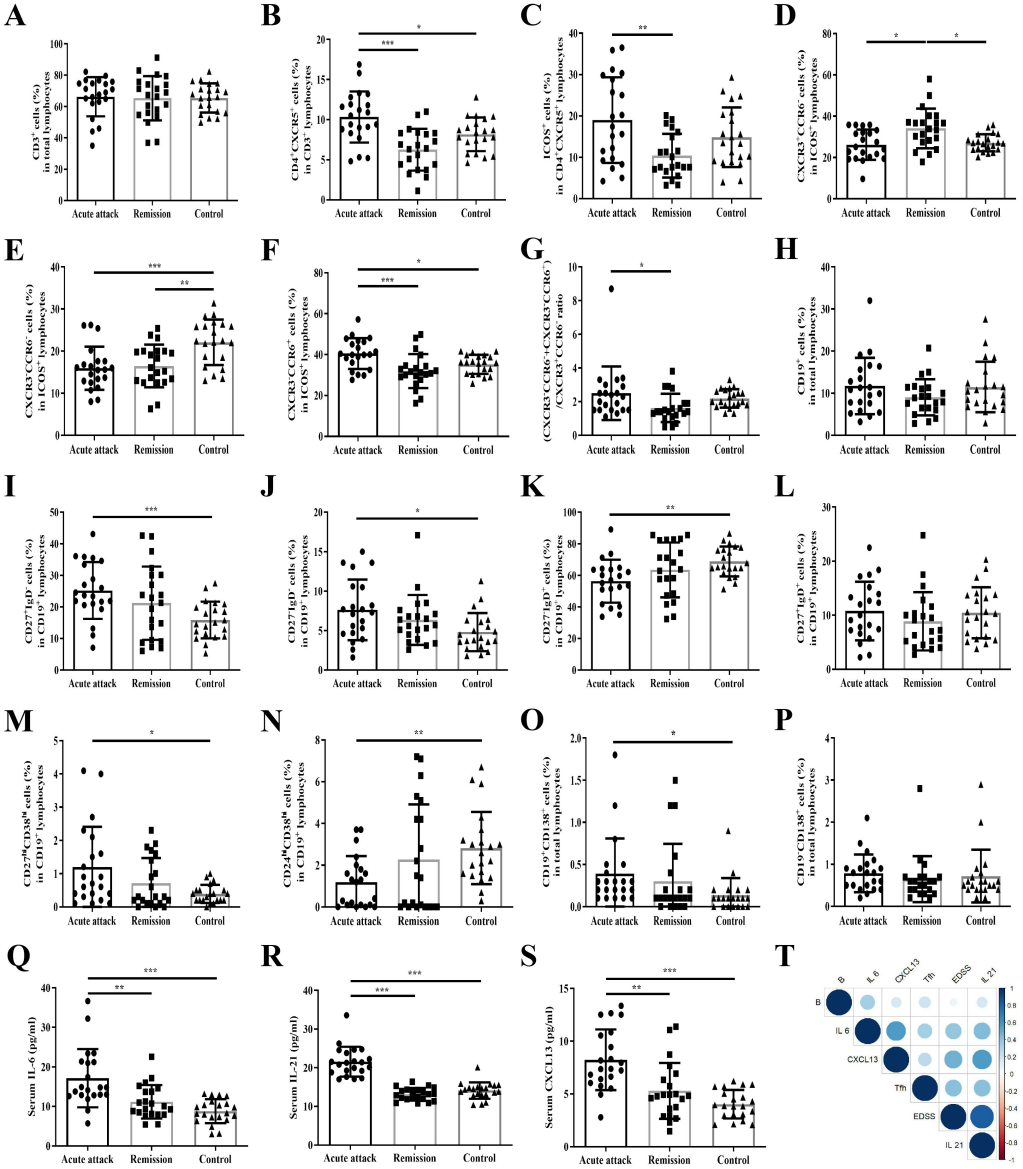
The proportions of Tfh and B cell subsets were dysregulated during acute attacks, accompanied by elevated IL-6, IL-21, and CXCL13 levels. **(A)** The proportion of CD3^+^ cells in total lymphocytes; **(B)** The proportion of CD4^+^CXCR5^+^ cells in CD3^+^ lymphocytes; **(C)** The proportion of ICOS^+^ cells in CD4^+^CXCR5^+^ lymphocytes; **(D)** The proportion of CXCR3^+^CCR6− cells in ICOS^+^ lymphocytes; **(E)** The proportion of CXCR3−CCR6− cells in ICOS^+^ lymphocytes; **(F)** The proportion of CXCR3−CCR6^+^ cells in ICOS^+^ lymphocytes; **(G)** The ratio of (CXCR3−CCR6− + CXCR3−CCR6^+^)/CXCR3^+^CCR6− cells; **(H)** The proportion of CD19^+^ cells in total lymphocytes; **(I)** The proportion of CD27^+^IgD− cells in CD19^+^ lymphocytes; **(J)** The proportion of CD27−IgD− cells in CD19^+^ lymphocytes; **(K)** The proportion of CD27−IgD^+^ cells in CD19^+^ lymphocytes; **(L)** The proportion of CD27^+^IgD^+^ cells in CD19^+^ lymphocytes; **(M)** The proportion of CD27^hi^CD38^hi^ cells in CD19^+^ lymphocytes; **(N)** The proportion of CD24^hi^CD38^hi^ cells in CD19^+^ lymphocytes; **(O)** The proportion of CD19^+^CD138^+^ cells in total lymphocytes; **(P)** The proportion of CD19−CD138^+^ cells in total lymphocytes; **(Q)** Serum IL-6 level; **(R)** Serum IL-21 level; **(S)** Serum CXCL13 level; **(T)** Correlation analysis of the above outcome measures including all patients (acute attack + remission). *P < 0.05, **P < 0.01, ***P < 0.001.

The gating strategy for circulating Tfh cell subsets is presented in **Supplementary Figure 1**. The frequency of CD4^+^CXCR5^+^ Tfh cells was significantly increased during acute attacks compared to both remission (P < 0.001) and healthy controls (P < 0.001 and P = 0.030, respectively). Within the CD4^+^CXCR5^+^ Tfh population, the proportion of ICOS^+^ cells was significantly higher during the acute attacks than during remission (P = 0.006). Among ICOS^+^ Tfh cells, the CXCR3−CCR6^+^ subset (aTfh17) was markedly elevated during acute attacks relative to both remission and healthy controls (P < 0.001 and P = 0.048, respectively), whereas the CXCR3^+^CCR6− (aTfh1) and CXCR3−CCR6− (aTfh2) cell subsets were significantly reduced compared to remission or healthy controls (P = 0.013 and P < 0.001, respectively). Furthermore, the ratio of (aTfh2 + aTfh17)/aTfh1 was significantly higher in the acute attack group than in the remission group (P = 0.029) (**Figures 1A–G**).

The gating strategy for circulating B cell subsets is illustrated in **Supplementary Figure 2**. During acute attacks, the frequencies of CD27^+^IgD−, CD27−IgD−, and CD27^hi^CD38^hi^ cell subsets within the CD19^+^ B cell population, as well as CD19^+^CD138^+^ short-lived plasma cells, were significantly elevated compared to healthy controls (P < 0.001, P = 0.016, P = 0.018, and P = 0.047, respectively). In contrast, the proportions of CD27−IgD+ and CD24^hi^CD38^hi^ subsets within CD19^+^ B cells were significantly lower than those in healthy controls (P = 0.004 and P = 0.003, respectively). No other B cell subsets showed statistically significant differences among the three groups (**Figures 1H–P**).

### Elevated serum levels of IL-6, IL-21 and CXCL13 during acute attacks and correlation analysis

Serum IL-6 levels were significantly higher during acute attacks compared with remission and healthy controls (P = 0.008 and P < 0.001, respectively). Similarly, IL-21 levels were markedly elevated during acute attacks relative to both remission and healthy controls (both P < 0.001). Serum CXCL13 levels were also significantly increased during the acute phase compared with remission and healthy controls (P < 0.001 and P = 0.004, respectively) (**Figures 1Q–S**).

Correlation analyses including all patients (acute attack and remission phases combined) are summarized in **Figure 1T**. EDSS scores showed positive correlations with the frequency of circulating CD4^+^CXCR5^+^ Tfh cells (R = 0.418, P = 0.006), as well as serum levels of IL-6 (R = 0.384, P = 0.012), IL-21 (R = 0.823, P < 0.001), and CXCL13 levels (R = 0.479, P = 0.001). In addition, the proportion of circulating CD4^+^CXCR5^+^ Tfh cells positively correlated with serum IL-6 (R = 0.331, P = 0.032) and IL-21 levels (R = 0.416, P = 0.006). The frequency of circulating CD19^+^ B cells also showed a positive correlation with serum IL-6 levels (R = 0.330, P = 0.033).

### Tfh cells promote B cells proliferation, differentiation, and AQP4-ab production

**Figure 2:**
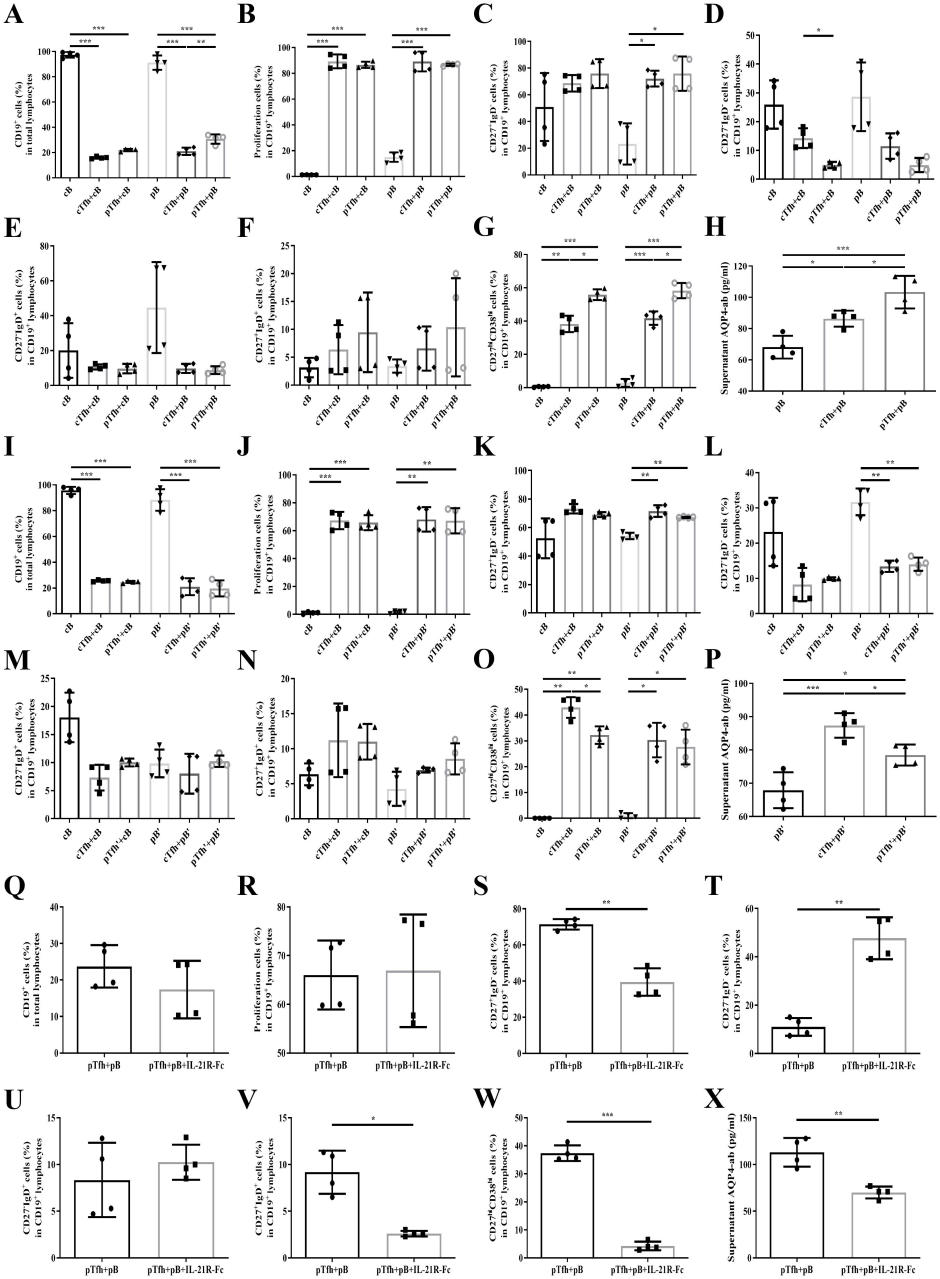
Tfh cells promoted B cell proliferation, differentiation, and AQP4-ab production. **(A–H)**. Sorting Tfh and B cells from healthy controls (cTfh, cB) or NMOSD patients in the acute attack (pTfh, pB); **(I–P)**. Sorting Tfh and B cells from healthy controls (cTfh, cB) or NMOSD patients in the remission (pTfh’, pB’); **(Q–X)**. Sorting Tfh and B cells from NMOSD patients in the acute attack (pTfh, pB) and adding IL-21R-Fc as the intervention; **(A, I, Q)**. The proportion of CD19^+^ cells in total lymphocytes; **(B, J, R)**. The proportion of proliferating cells in CD19^+^ lymphocytes; **(C, K, S)**. The proportion of CD27^+^IgD− cells in CD19^+^ lymphocytes; **(D, L, T)**. The proportion of CD27−IgD− cells in CD19^+^ lymphocytes; **(E, M, U)**. The proportion of CD27−IgD^+^ cells in CD19^+^ lymphocytes; **(F, N, V)**. The proportion of CD27^+^IgD^+^ cells in CD19^+^ lymphocytes; **(G, O, W)**. The proportion of CD27^hi^CD38^hi^ cells in CD19^+^ lymphocytes; **(H, P, X)**. Supernatant AQP4-ab level. *P < 0.05, **P < 0.01, ***P < 0.001.

The gating strategies for cell sorting and flow cytometric analysis are shown in **Supplementary Figures 3, 4**, and participant characteristics are detailed in **Supplementary Table 3 (Panel 1A–C)**. In co-culture experiments, Tfh cells significantly enhanced B cell proliferation, increased the proportion of CD27^hi^CD38^hi^ plasmablasts within the CD19^+^ B cell population, and promoted AQP4-ab production compared with B cells cultured alone (all P < 0.001) (**Figures 2A–H**). Notably, B cells isolated from patients during the acute attacks (pB) exhibited baseline proliferation (15.1%) even under monoculture conditions, whereas B cells from patients in remission (pB′) or healthy controls (cB) showed minimal proliferation. Tfh cells from acute-phase patients (pTfh) demonstrated greater efficacy than those from healthy controls (cTfh) in promoting plasmablast differentiation and AQP4-ab production (P = 0.013 and P = 0.034, respectively). In contrast, Tfh cells from patients in remission (pTfh′) exhibited a significantly reduced capacity to support these processes compared with cTfh (P = 0.048 and P = 0.036, respectively) (**Figures 2I–P**).

In the Tfh–B cell co-culture system, treatment with IL-21R–Fc significantly suppressed the frequencies of CD27^+^IgD^-^, CD27^-^IgD^-^, CD27^+^IgD^+^, and CD27^hi^CD38^hi^ subsets within the CD19^+^ B cell population (P = 0.002, P = 0.001, P = 0.010, and P < 0.001, respectively) and reduced AQP4-ab production (P = 0.007) (**Figures 2Q–X**).

### IL-6 promotes the differentiation and function of Tfh and B cells

**Figure 3:**
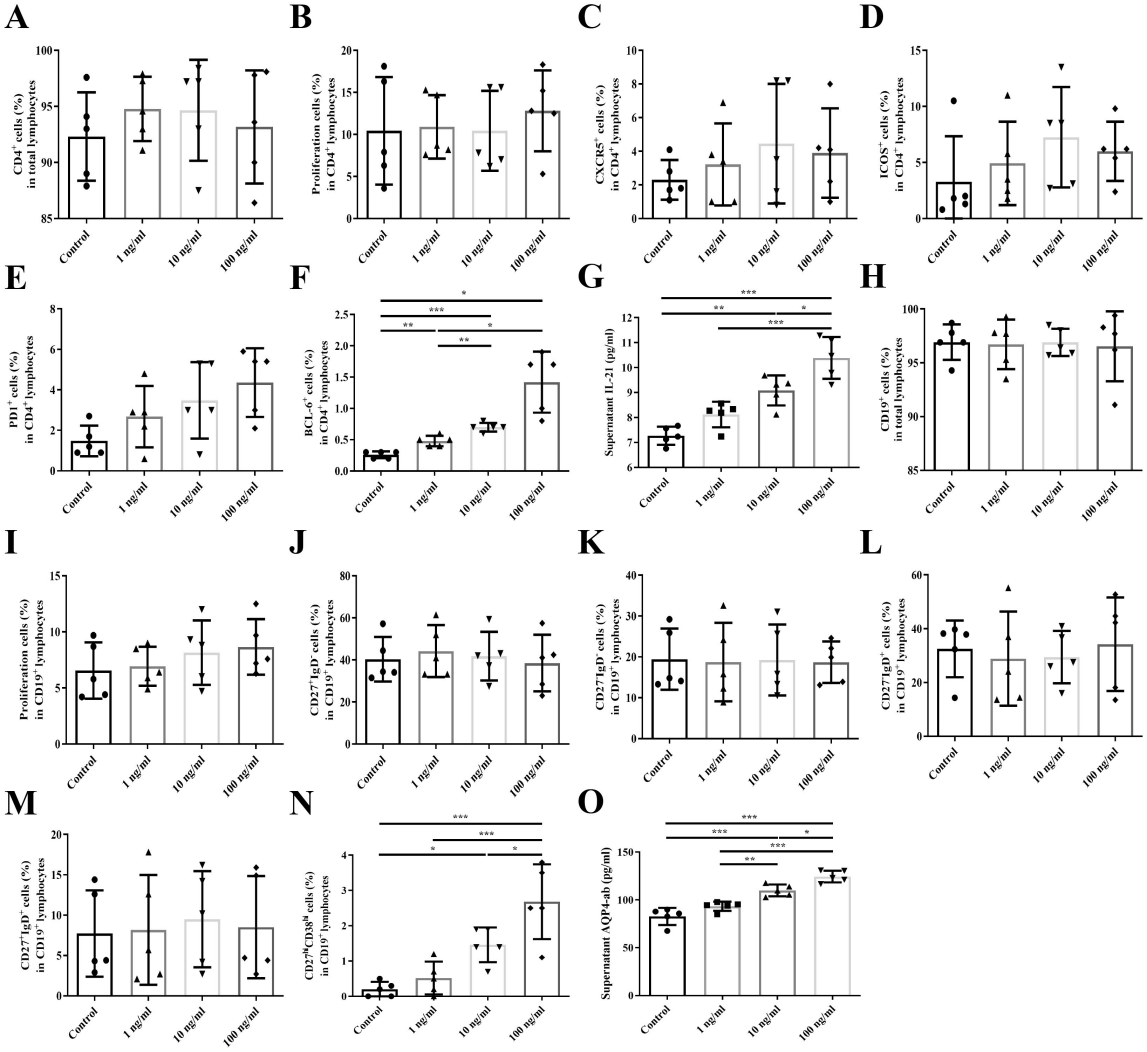
IL-6 promoted the differentiation and function of Tfh and B cells. **(A)** The proportion of CD4^+^ cells in total lymphocytes; **(B)** The proportion of proliferating cells in CD4^+^ lymphocytes; **(C)** The proportion of CXCR5 in CD4^+^ lymphocytes; **(D)** The proportion of ICOS in CD4^+^ lymphocytes; **(E)** The proportion of PD-1 in CD4^+^ lymphocytes; **(F)** The proportion of BCL6 in CD4^+^ lymphocytes; **(G)** Supernatant IL-21 level; **(H)** The proportion of CD19^+^ cells in total lymphocytes; **(I)** The proportion of proliferating cells in CD19^+^ lymphocytes; **(J)** The proportion of CD27^+^IgD− cells in CD19^+^ lymphocytes; **(K)** The proportion of CD27−IgD− cells in CD19^+^ lymphocytes; **(L)**. The proportion of CD27−IgD^+^ cells in CD19^+^ lymphocytes; **(M)**. The proportion of CD27^+^IgD^+^ cells in CD19^+^ lymphocytes; **(N)**. The proportion of CD27^hi^CD38^hi^ cells in CD19^+^ lymphocytes; **(O)**. Supernatant AQP4-ab level. *P < 0.05, **P < 0.01, ***P < 0.001.

The gating strategies for cell sorting and flow cytometric analysis are detailed in **Supplementary Figures 3, 5, 6**, and participant characteristics are presented in **Supplementary Table 3 (Panel 2A, B)**. The frequency of BCL6 expression in Tfh cells increased significantly with escalating concentrations of IL-6 (P < 0.001). No significant differences were observed among groups for other T cell phenotypes, including the proportions of CD4, proliferating cells, CXCR5, ICOS, and PD-1 within the CD4^+^ T cell population. Correspondingly, IL-21 levels in the culture supernatant increased significantly with rising IL-6 concentrations (P < 0.001) (**Figures 3A–G**).

Similarly, the proportion of CD27^hi^CD38^hi^ plasmablasts within the CD19^+^ B cell population increased significantly with increasing IL-6 concentration (P < 0.001). Other B cell phenotypes—including total CD19+ B cells, proliferating cells, and subsets defined by CD27 and IgD expression (CD27^+^IgD−, CD27−IgD−, CD27−IgD+, and CD27^+^IgD^+^)—did not show significant changes. In addition, AQP4-ab levels in the culture supernatant were significantly upregulated in response to increasing IL-6 concentrations (P < 0.001) (**Figures 3H–O**).

### Differential effects of IL-6R-Fc and IL-21R-Fc on Tfh and B cells

**Figure 4:**
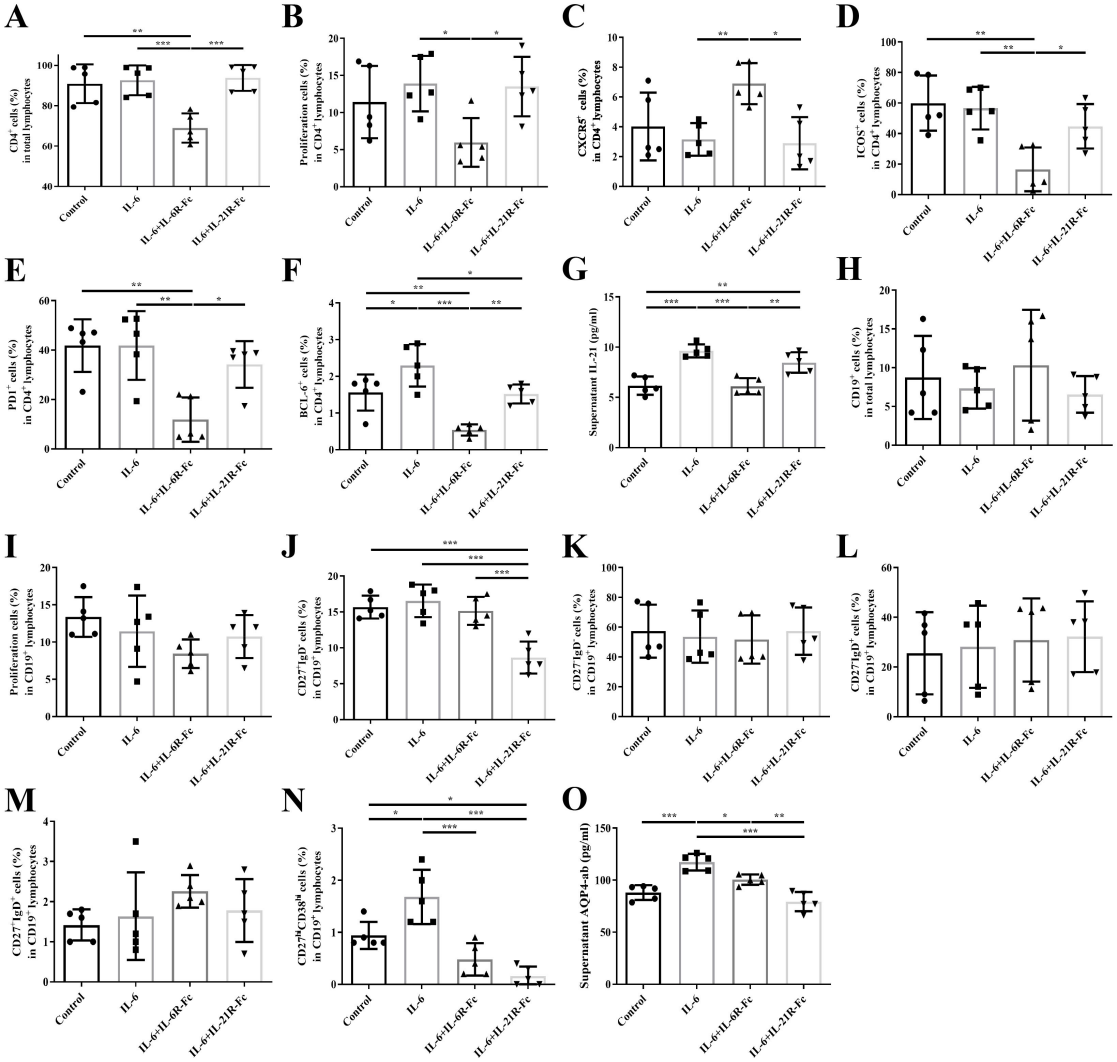
The differential effects of IL-6/21R-Fc on Tfh and B cells. **(A)** The proportion of CD4^+^ cells in total lymphocytes; **(B)** The proportion of proliferating cells in CD4^+^ lymphocytes; **(C)** The proportion of CXCR5 in CD4^+^ lymphocytes; **(D)** The proportion of ICOS in CD4^+^ lymphocytes; **(E)** The proportion of PD-1 in CD4^+^ lymphocytes; **(F)** The proportion of BCL6 in CD4^+^ lymphocytes; **(G)** Supernatant IL-21 level; **(H)** The proportion of CD19^+^ cells in total lymphocytes; **(I)** The proportion of proliferating cells in CD19^+^ lymphocytes; **(J)** The proportion of CD27^+^IgD− cells in CD19^+^ lymphocytes; **(K)** The proportion of CD27−IgD− cells in CD19^+^ lymphocytes; **(L)**. The proportion of CD27−IgD^+^ cells in CD19^+^ lymphocytes; **(M)**. The proportion of CD27^+^IgD^+^ cells in CD19^+^ lymphocytes; **(N)**. The proportion of CD27^hi^CD38^hi^ cells in CD19^+^ lymphocytes; **(O)**. Supernatant AQP4-ab level. *P < 0.05, **P < 0.01, ***P < 0.001.

The gating strategies for cell sorting and flow cytometric analysis are provided in **Supplementary Figures 3, 7, 8**, and participant profiles are summarized in **Supplementary Table 3 (Panel 3A, B)**. In Tfh cells, treatment with IL-6R–Fc significantly suppressed the proportions of CD4+ cells, proliferating cells, ICOS, PD-1, and BCL6 expression within the CD4^+^ T cell population, and reduced IL-21 levels in the culture supernatant (P < 0.001, P = 0.030, P = 0.004, P = 0.003, P < 0.001, and P < 0.001, respectively). In contrast, IL-6R–Fc treatment significantly increased the proportion of CXCR5 within the CD4^+^ T cell population (P = 0.007). Treatment with IL-21R–Fc resulted in a reduction only in BCL6 expression (P = 0.037). Compared with IL-21R–Fc, IL-6R–Fc induced a more pronounced downregulation of BCL6 (P = 0.008) (**Figures 4A–G**).

With respect to B cells, IL-6R–Fc markedly inhibited the frequency of CD27^hi^CD38^hi^ plasmablasts within the CD19^+^ B cell population and reduced AQP4-ab levels in the culture supernatant (P < 0.001 and P = 0.013, respectively). IL-21R–Fc significantly decreased the proportions of CD27^+^IgD− and CD27^hi^CD38^hi^ B cell subsets and suppressed AQP4-ab production (all P < 0.001). Notably, IL-6R–Fc resulted in a greater reduction in AQP4-ab levels than IL-21R–Fc (P = 0.002) (**Figures 4H–O**).

### B cell subsets exert regulatory effects on Tfh cells

**Figure 5:**
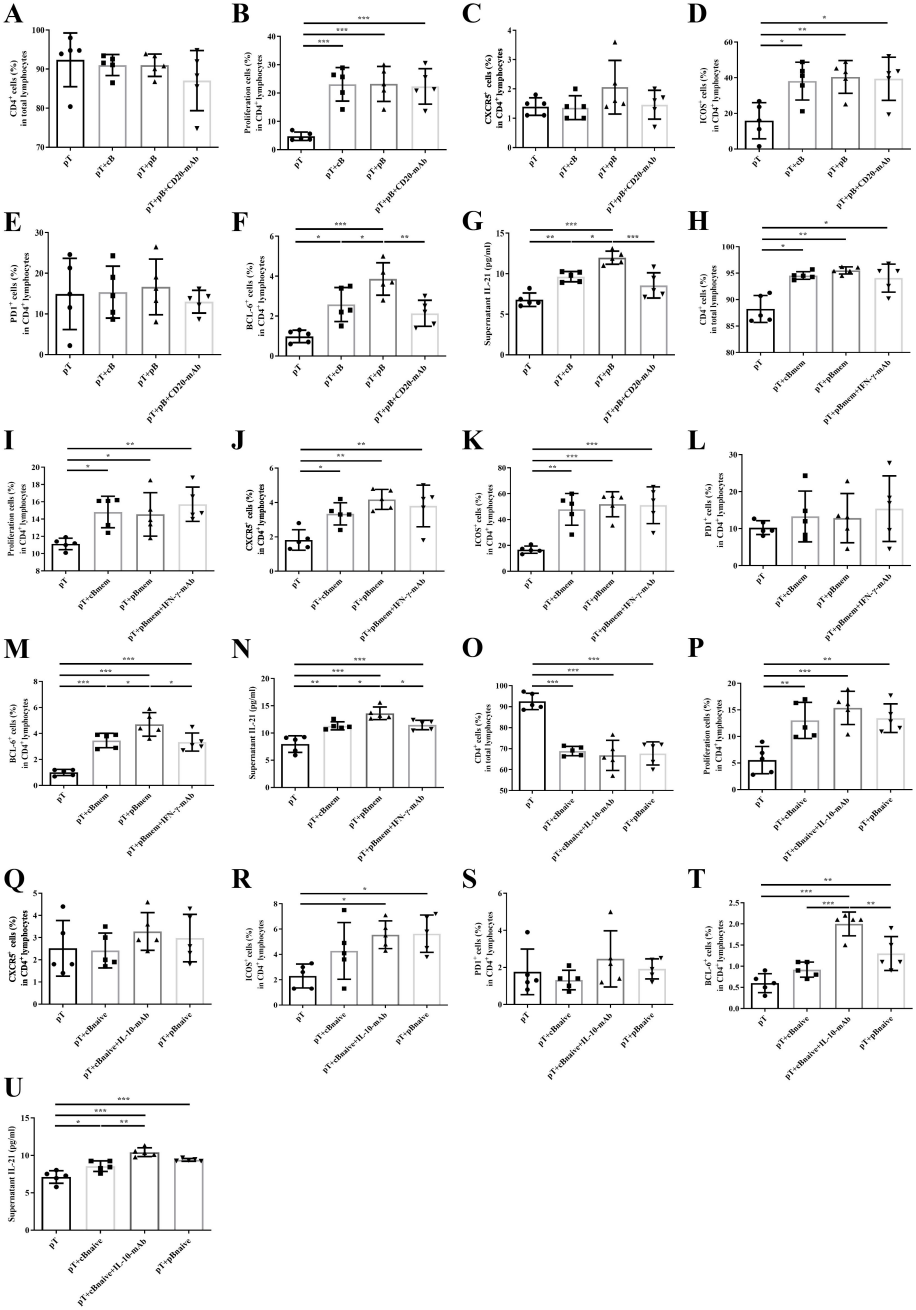
B cell subsets had counteraction on Tfh cells. **(A–G)**. Sorting T and B cells from healthy controls (cB) or NMOSD patients in the acute attack (pT, pB) and adding anti-CD20 monoclonal antibody as the intervention; **(H–N)**. Sorting T and memory B cells from healthy controls (Bmem) or NMOSD patients in the acute attack (pT, pBmem) and adding anti-IFN-γ monoclonal antibody as the intervention; **(O–U)**. Sorting T and naive B cells from healthy controls (cBaive) or NMOSD patients in the acute attack (pT, pBaive) and adding anti–IL-10 monoclonal antibody as the intervention; **(A, H, O)**. The proportion of CD4^+^ cells in total lymphocytes; **(B, I, P)**. The proportion of proliferating cells in CD4^+^ lymphocytes; **(C, J, Q)**. The proportion of CXCR5 in CD4^+^ lymphocytes; **(D, K, R)**. The proportion of ICOS in CD4^+^ lymphocytes; **(E, L, S)**. The proportion of PD-1 in CD4^+^ lymphocytes; **(F, M, T)**. The proportion of BCL6 in CD4^+^ lymphocytes; **(G, N, U)**. Supernatant IL-21 level. *P < 0.05, **P < 0.01, ***P < 0.001.

The gating strategies for cell sorting and flow cytometric analysis are shown in **Supplementary Figures 3, 7, 9**, and participant characteristics are detailed in **Supplementary Table 3 (Panel 4A–C)**. CD19^+^ B cells significantly enhanced Tfh cell proliferation and upregulated the expression of ICOS and BCL6 expression within the CD4^+^ T cell population (P < 0.001, P = 0.010, and P < 0.001, respectively). B cells isolated from patients (pB) were more effective than those from healthy controls (cB) in promoting BCL6 expression and IL-21 production (P = 0.044 and P = 0.011, respectively), and these effects were reversed by treatment with anti-CD20 monoclonal antibody (P = 0.006 and P < 0.001, respectively) (**Figures 5A–G**).

CD19^+^CD27^+^ memory B cells increased the proportions of CD4+ cells, proliferating cells, CXCR5, ICOS, and BCL6 within the CD4^+^ T cell population (P = 0.008, P = 0.047, P = 0.001, P < 0.001, and P < 0.001, respectively). Patient-derived memory B cells (pBmem) exhibited a stronger capacity to promote of BCL6 expression and IL-21 production than those from healthy controls (cBmem) (P = 0.035 and P = 0.022, respectively), and these effects were abrogated by anti–interferon-γ (IFN-γ) monoclonal antibody treatment (P = 0.020 and P = 0.034, respectively) (**Figures 5H–N**).

CD19^+^CD27− naive B cells enhanced Tfh cell proliferation (P = 0.005) but reduced the proportion of CD4+ cells (P < 0.001). Compared with patient-derived naive B (pBnaive) cells (pB_naive), healthy naive B cells (cB_naive) exhibited inhibitory effects on BCL6 expression and IL-21 production, which were reversed by anti–IL-10 monoclonal antibody treatment (P < 0.001 and P = 0.001, respectively) (**Figures 5O–U**).

## Discussion

In this study, we demonstrated that circulating Tfh and B cell subsets are dysregulated in AQP4-ab–positive NMOSD during acute attacks, accompanied by elevated levels of IL-6, IL-21, and CXCL13. Expanded Disability Status Scale (EDSS) scores showed a positive correlations with the frequency of circulating total Tfh cells and serum concentrations of IL-6, IL-21, and CXCL13. A reciprocal interaction exists between Tfh and B cells: Tfh cells promote B cell proliferation, differentiation, and AQP4-ab production, whereas B cell subsets enhance Tfh cell proliferation, differentiation, and IL-21 secretion. This latter effect is inhibited by anti-CD20 and anti–interferon-γ (IFN-γ) monoclonal antibodies, but enhanced by anti–IL-10 monoclonal antibody.

Tfh cells are characterized by the expression of CXCR5, ICOS, PD-1, and CD40L, the transcription factor BCL6, and the secretion of IL-21 (15). ICOS expression distinguishes activated from quiescent Tfh cells, whereas the combination of CXCR3 and CCR6 defines three major subsets: CXCR3^+^CCR6− (Tfh1), CXCR3−CCR6− (Tfh2), and CXCR3−CCR6^+^ (Tfh17) cell subsets (16). Our findings confirmed an increased frequency of circulating Tfh cells and the activated Tfh17 subset during acute attacks—cell populations considered to be highly pathogenic and particularly effective in supporting B cell responses (17). Notably, Tfh cell activity during remission phase was even lower than that observed in healthy controls, which may be attributed to the prolonged use of oral immunosuppressive agents.

B cells and humoral immunity play a pivotal role in the pathogenesis of NMOSD (18). Previous studies have reported dysregulation of B cell subsets in NMOSD, including switched memory B cells, double-negative B cells, and plasmablasts, which may serve as biomarkers of disease activity (19–21). Our results further revealed a reduced proportion of naive B cells and transitional regulatory B cells, a deficit that may be partially reversed by immunosuppressive therapy. Plasmablasts and plasma cells are the primary sources of AQP4-ab, and IL-6 has been shown to promote AQP4-ab production by these cells (21). Elevated levels of IL-6, IL-21, and CXCL13 have been previously documented in NMOSD and are consistent with our observations. CXCL13, also known as B lymphocyte chemoattractant, is predominantly secreted by activated T helper cells—including Tfh cells—and antigen-presenting cells (22–24). As the sole known ligand for CXCR5, which is expressed on mature B cells and Tfh cells, aberrant CXCL13 expression of CXCL13 in ectopic germinal centers has been implicated in various autoimmune diseases (25). Furthermore, CXCL13 facilitates the migration of B cells to inflammatory sites within the central nervous system (26). Previous studies have shown that both serum and cerebrospinal fluid levels of CXCL13 are elevated in NMOSD patients compared with those with multiple sclerosis and healthy controls (6, 27–29), potentially promoting neuroinflammation by recruiting antibody-secreting B cells.

*In vitro* co-culture experiments demonstrated that Tfh cells significantly enhance B cell proliferation, differentiation, and AQP4-ab secretion, consistent with previous reports (11). Notably, the functional capacity of Tfh cells during acute attacks was greater than that observed during remission or in healthy controls, whereas Tfh cells from patients in remission exhibited diminished activity. These results align with prior findings in myasthenia gravis and may be explained by the altered distributions of Tfh cell subsets in these conditions (30). Following IL-21R–Fc intervention, the frequency of plasmablasts and AQP4-ab levels were significantly reduced, corroborating previous studies in NMOSD and myasthenia gravis (7, 15, 30). It has been established that IL-21R–Fc does not suppress general T helper cell activation but specifically blocks T cell–mediated B cell activation and plasma cell differentiation (10).

We further observed that IL-6 enhances BCL6 expression in Tfh cells and promotes IL-21 secretion, without affecting other surface markers. These findings support previous evidence that IL-6 drives early Tfh cell differentiation and IL-21 production by inducing BCL6 expression (31, 32). In B cells, IL-6 selectively increased the proportion of plasmablasts and stimulated AQP4-ab secretion, with no significant impact on other B cell subsets. This is consistent with prior reports demonstrating that IL-6 supports plasmablast survival and antibody production, and that IL-6R is predominantly expressed on plasmablasts among B cell populations (21). These effects were further confirmed in dose-dependent experiments. It should be noted, however, that the 100 ng/ml concentration of IL-6 used exceeds physiological levels, suggesting that future studies should examine the biological relevance of lower, more physiologically representative doses.

Given that IL-6R and IL-21R are expressed on both Tfh cells and B cells, we investigated their functional contributions in these cell populations. Compared with IL-21R–Fc, IL-6R–Fc exerted a more pronounced inhibitory effect on the expression of Tfh cell surface marker expression and IL-21 production. Previous studies have demonstrated that BCL6 expression is regulated by both IL-6 and IL-21, consistent with our findings (31). These results suggest that IL-6 promotes Tfh cell differentiation and enhances IL-21 secretion—effects predominantly suppressed by IL-6R–Fc—whereas IL-21R–Fc exerts a relatively weaker inhibitory effect on Tfh cells. Notably, IL-21R–Fc has been shown that IL-21R-Fc does not to inhibit T helper cell activation but to selectively block T cell–mediated B cell activation and plasma cell differentiation induced by Th cells (10). In contrast, IL-21R–Fc demonstrated a stronger inhibitory effect on B cell subsets and AQP4-ab production compared with IL-6R–Fc. During T–B cell collaboration, IL-21 is essential for IL-6–mediated immunoglobulin production (32). Given that IL-6R is predominantly expressed on plasmablasts within the B cell compartment, its blockade markedly reduces plasmablast frequency without altering other B cell subsets (21). Collectively, these observations indicate that IL-6 contributes to B cell differentiation and AQP4-ab production, effects primarily attenuated by IL-21R–Fc, whereas IL-6R–Fc shows a comparatively limited inhibitory capacity in B cells. Tocilizumab, a monoclonal antibody targeting IL-6R, has been shown to significantly reduce relapse rates in NMOSD, as well as decrease the frequencies of Tfh cells and plasma cells, and restore imbalances among B cell subsets, thereby validating IL-6R as a key therapeutic target in NMOSD (19, 33).

We further examined the regulatory influence of B cells and their subsets on Tfh cells. Prior studies using co-culture systems of B cells and CD4^+^ T cells have indicated that regulatory B cells can suppress Tfh cell differentiation, whereas unstimulated B cells do not exert such inhibition (34). This discrepancy may arise from our use of CD19^+^CD27− naive B cells as putative regulatory B cells, whose phenotype may differ from that of truly unstimulated B cells, potentially leading to divergent experimental outcomes. Since Tfh cells lack CD20 expression, treatment with anti-CD20 monoclonal antibody effectively abrogated the observed promoting effects. Consistent with previous reports, rituximab administration has been associated with significant reductions in Tfh cell frequency and in the secretion of IL-21 and CXCL13 (35, 36). The differences between our findings and prior reports may be attributed to the *in vitro* nature of the current study, in which anti-CD20 monoclonal antibody may disrupt B–Tfh cellular interactions through functional blockade.

**Figure 6:**
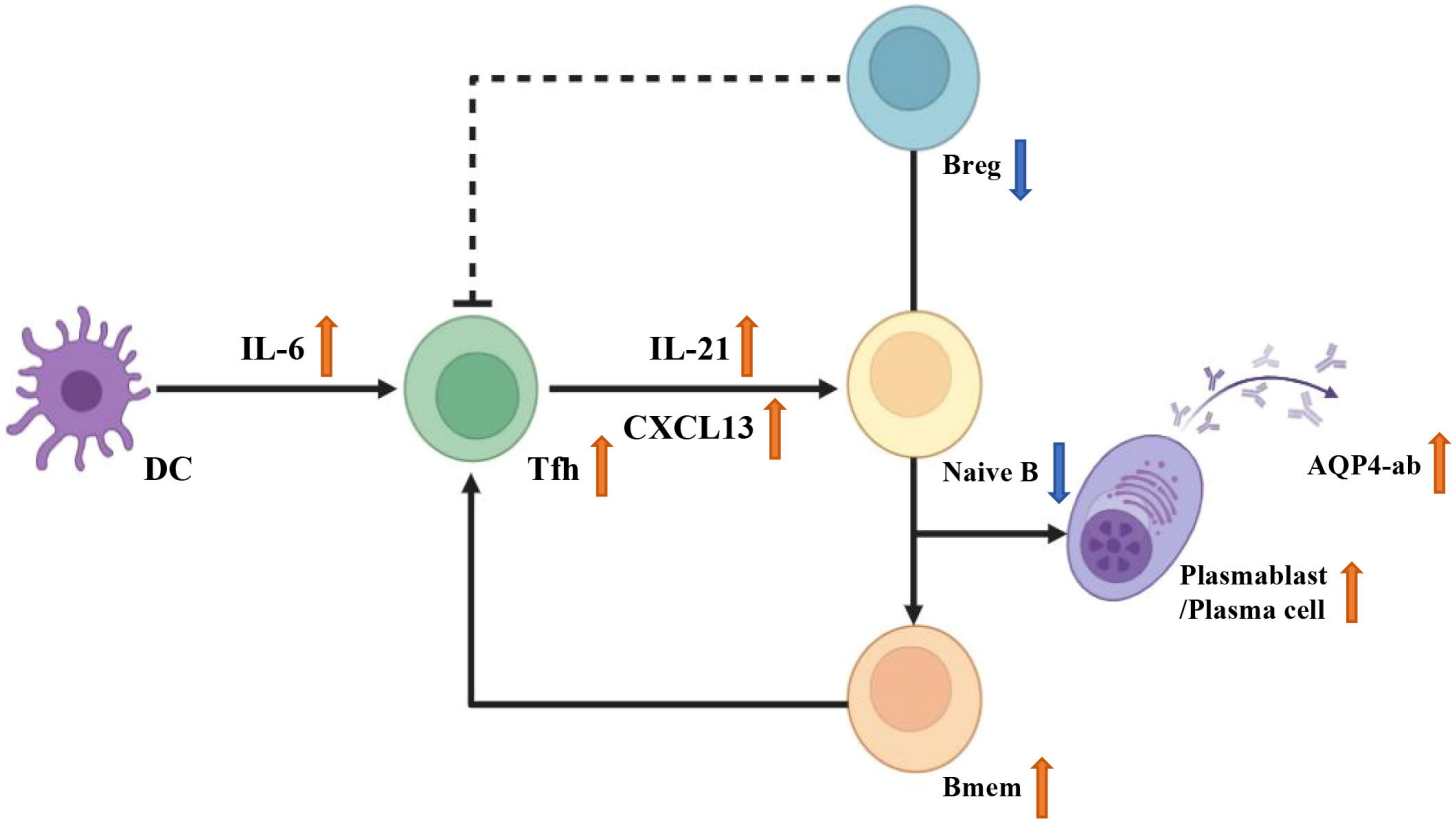
The IL-6-Tfh-B cell axis in NMOSD. IL-6 secreted by dendritic cells promotes the differentiation of naive T cells into Tfh cells. Tfh-derived IL-21 and CXCL13 induce the differentiation of naive B cells into memory B cells, plasmablasts and plasma cells, promote AQP4-ab production and chemotactic capacity. The differentiation of Tfh cells is promoted by memory B cells but inhibited by regulatory B cells. The cell icons in Image 6 were created using BioRender.com.

Previous evidence suggests that memory B cells secrete higher levels of interferon-γ (IFN-γ), whereas naive B cells produce more interleukin-10 (IL-10) (4). By neutralizing these cytokines using anti–IFN-γ or anti–IL-10 monoclonal antibodies, respectively, we observed alterations in BCL6 expression and IL-21 levels in the supernatant, highlighting the pro-inflammatory role of IFN-γ and the anti-inflammatory function of IL-10. Collectively, these findings support the notion that the IL-6–Tfh–B cell axis plays a critical role in the pathogenesis of AQP4-ab–positive NMOSD (**Figure 6**).

This study has several limitations. First, the sample size of the experimental groups was relatively small, which may have limited the statistical power to detect significant differences between groups. Second, CD4^+^ T cells rather than naive T cells were isolated, implying that the detected IL-21 in the supernatant may not have originated exclusively from Tfh cells. Third, some patients in the acute phase had received prior immunomodulatory therapy, which could have influenced the observed immune parameters. Finally, the number of subjects included in the functional experiments was limited to four to five.

## Conclusions

In AQP4-ab–positive NMOSD, circulating Tfh and B cell subsets are dysregulated and accompanied by elevated levels of IL-6, IL-21, and CXCL13. There is a reciprocal interaction between Tfh and B cells: Tfh cells promote B cell proliferation, differentiation, and AQP4-ab production, while distinct B cell subsets reciprocally enhance Tfh cell expansion, differentiation, and IL-21 secretion.

## Data Availability

The original contributions presented in the study are included in the article/**Supplementary Material**. Further inquiries can be directed to the corresponding authors.
