# Supplementary material for: Dysregulated Tfh/B cells and their interactions in neuromyelitis optica spectrum disorder
Source: Front Immunol. 2026 Jan 6;16:1704282. doi: 10.3389/fimmu.2025.1704282 (PMC12815817; doi:10.3389/fimmu.2025.1704282)
Supplement: Supplementary file 1 [file DataSheet1.docx]

**Supplement file**

| **Supplementary Table 1. Flow panels included in the study** | | |
| --- | --- | --- |
| Panel | Lymphocyte subsets | Combinations of monoclonal antibody |
| Panel T | CD3^+^CD4^+^CXCR5^+^ follicular helper T cell | CD3-BV605, CD4-PerCP-Cy5.5, CXCR5-PE-Cy7 |
|  | CD3^+^CD4^+^CXCR5^+^ICOS^+^ activated follicular helper T cell | CD3-BV605, CD4-PerCP-Cy5.5, CXCR5-PE-Cy7, ICOS-APC |
|  | CD3^+^CD4^+^CXCR5^+^ICOS^+^CXCR3^+^CCR6^-^ activated follicular helper T cell 1 | CD3-BV605, CD4-PerCP-Cy5.5, CXCR5-  PE-Cy7, ICOS-APC, CXCR3-PE, CCR6-FITC |
|  | CD3^+^CD4^+^CXCR5^+^ICOS^+^CXCR3^-^CCR6^-^ activated follicular helper T cell 2 | CD3-BV605, CD4 PerCP-Cy5.5, CXCR5-  PE-Cy7, ICOS APC, CXCR3-PE, CCR6-FITC |
|  | CD3^+^CD4^+^CXCR5^+^ICOS^+^CXCR3^-^CCR6^+^ activated follicular helper T cell 17 | CD3-BV605, CD4-PerCP-Cy5.5, CXCR5-  PE-Cy7, ICOS-APC, CXCR3-PE, CCR6-FITC |
| Panel B | CD19^+^CD27^+^IgD^-^ switched memory B cell | CD19-PE-Cy7, CD27-FITC, IgD-BV510 |
|  | CD19^+^CD27^+^IgD^-^ double negative B cell | CD19-PE-Cy7, CD27-FITC, IgD-BV510 |
|  | CD19^+^CD27^-^IgD^+^ naive B cell | CD19-PE-Cy7, CD27-FITC, IgD-BV510 |
|  | CD19^+^CD27^+^IgD^+^ unswitched memory B cell | CD19-PE-Cy7, CD27-FITC, IgD-BV510 |
|  | CD19^+^CD27^hi^CD38^hi^ plasmablasts | CD19-PE-Cy7, CD27-FITC, CD38-APC |
|  | CD19^+^CD24^hi^CD38^hi^ regulatory B cell | CD19-PE-Cy7, CD24-PE, CD38-APC |
|  | CD19^+^CD138^+^ short-lived plasma cell | CD19-PE-Cy7, CD138-BV421 |
|  | CD19^-^CD138^+^ long-lived plasma cell | CD19-PE-Cy7, CD138-BV421 |

| **Supplementary Table 2. Flow product details included in the study** | | | | | |
| --- | --- | --- | --- | --- | --- |
| Monoclonal antibody | Fluorophore | Clone | Catalog number | Brand | Panel |
| CD3 | BV605 | OKT3 | 317322 | Biolegend | Panel T |
| CD4 | PerCP-Cy5.5 | RPA-T4 | 45-0049-42 | eBioscience | Panel T, 1A-C, 2A, 3A-B, 4A-C |
| CXCR5 | PE-Cy7 | MU5UBEE | 25-9185-42 | eBioscience | Panel T |
| CXCR5 | PE | MU5UBEE | 12-9185-42 | eBioscience | Panel 1A-C |
| CXCR5 | BV605 | J252D4 | 356930 | Biolegend | Panel 2-3A, 4A-C |
| ICOS | APC | ISA-3 | 17-9948-42 | eBioscience | Panel T, 2-4A |
| ICOS | BV510 | C398.4A | 313525 | Biolegend | Panel 4B-C |
| CXCR3 | PE | CEW33D | 12-1839-42 | eBioscience | Panel T |
| CCR6 | FITC | G034E3 | 353412 | Biolegend | Panel T |
| CD19 | PE-Cy7 | HIB19 | 25-0199-42 | eBioscience | Panel B, 1A-C, 2B, 3A-B, 4A-C |
| CD27 | FITC | O323 | 11-0279-42 | eBioscience | Panel B |
| CD27 | BV421 | O323 | 302824 | Biolegend | Panel 1A-C, 2-3B |
| CD27 | APC | O323 | 17-0279-42 | eBioscience | Panel 4B-C |
| IgD | BV510 | IA6-2 | 348219 | Biolegend | Panel B, 1A-C, 2-3B |
| CD24 | PE | SN3 A5-2H10 | 12-0247-41 | eBioscience | Panel B |
| CD38 | APC | HIT2 | 303510 | Biolegend | Panel B, 1A-C, 2-3B |
| CD138 | BV421 | MI15 | 356516 | Biolegend | Panel B |
| PD1 | BV421 | EH12.2H7 | 329920 | Biolegend | Panel 2-3A, 4A-C |
| BCL6 | PE | K112-91 | 561522 | BD Biosciences | Panel 2-3A, 4A-C |
| Viability Dye 780 | — | — | 62910-00 | Peprotech | Panel 1A-C, Panle 2A-B, Panle 3A-B, Panel 4A-C |
| CFSE | — | — | 63310-00 | Peprotech | Panel 1A-C, Panle 2A-B, Panle 3A-B, Panel 4A-C |

| **Supplementary Table 3. The profiles of AQP4-ab-positive NMOSD patients and control subjects used in the co-culture experiments** | | | | | |
| --- | --- | --- | --- | --- | --- |
| Panel | Characteristics | Acute attack | Remission | Control group | P value |
| Panel 1A (N=4/4) | Female (%) | 4 (100) | — | 4 (100) | 1.000 |
|  | Age at recruitment, y | 40.6 ± 7.2 | — | 38.7 ± 5.0 | 0.670 |
| Panel 1B (N=4/4) | Female (%) | — | 3 (75) | 3 (75) | 1.000 |
|  | Age at recruitment, y | — | 39.3 ± 4.4 | 36.2 ± 5.6 | 0.416 |
| Panel 1C (N=4) | Female (%) | 4 (100) | — | — | — |
|  | Age at recruitment, y | 54.7 ± 8.8 | — | — | — |
| Panel 2A (N=5) | Female (%) | 5 (100) | — | — | — |
|  | Age at recruitment, y | 54.9 ± 5.3 | — | — | — |
| Panel 2B (N=5) | Female (%) | 5 (100) | — | — | — |
|  | Age at recruitment, y | 54.9 ± 5.3 | — | — | — |
| Panel 3A (N=5) | Female (%) | 5 (100) | — | — | — |
|  | Age at recruitment, y | 32.8 ± 7.5 | — | — | — |
| Panel 3B (N=5) | Female (%) | 5 (100) | — | — | — |
|  | Age at recruitment, y | 32.8 ± 7.5 | — | — | — |
| Panel 4A (N=5/5) | Female (%) | 5 (100) | — | 4 (80) | 1.000 |
|  | Age at recruitment, y | 48.0 ± 12.5 | — | 50.6 ± 11.5 | 0.745 |
| Panel 4B (N=5/5) | Female (%) | 4 (80) | — | 4 (80) | 1.000 |
|  | Age at recruitment, y | 44.3 ± 6.2 | — | 42.9 ± 10.4 | 0.800 |
| Panel 4C (N=5/5) | Female (%) | 4 (80) | — | 4 (80) | 1.000 |
|  | Age at recruitment, y | 33.7 ± 4.0 | — | 35.5 ± 10.1 | 0.723 |

**Supplementary Figure 1. Gating strategy for circulating Tfh cell subsets.** A: Gating the single cell population; B: Gating the lymphocyte population; C: Gating the CD3^+^ T cells from the lymphocyte population; D: Gating the CD4^+^CXCR5^+^ Tfh cells from the CD3^+^ T cell population; E: Gating the ICOS^+^ cells from the CD4^+^CXCR5^+^ Tfh cell population; F: ICOS isotype control; G: Gating the CXCR3^+^CCR6^-^ cells in the upper left quadrant, CXCR3^-^CCR6^-^ cells in the lower left quadrant, and CXCR3^-^CCR6^+^ cells in the lower right quadrant from the ICOS^+^ cell population.


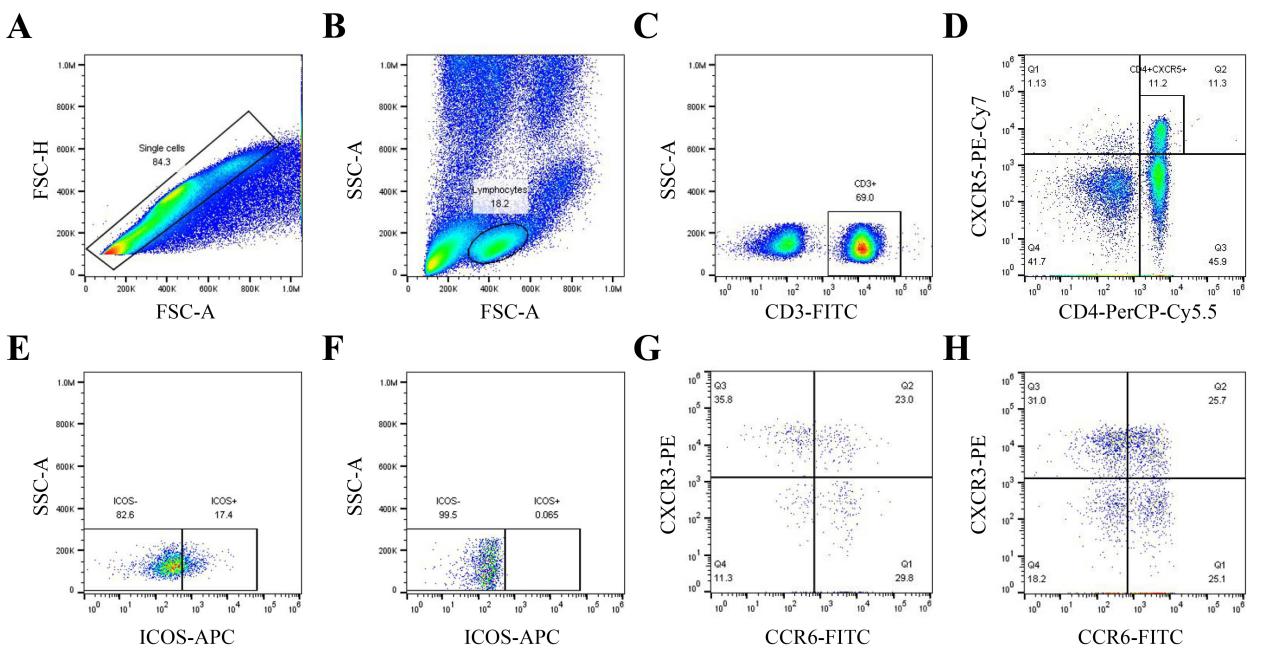


**Supplementary Figure 2. Gating strategy for circulating B cell subsets.** A: Gating the single cell population; B: Gating the lymphocyte population; C: Gating the CD19^+^ B cells from the lymphocyte population; D: Gating the CD27^+^IgD^-^ cells in the upper left quadrant, CD27^-^IgD^-^ cells in the lower left quadrant, CD27^-^IgD^+^ cells in the lower right quadrant, CD27^+^IgD^+^ cells in the upper right quadrant from the CD19^+^ B cell population; E: Gating the CD27^hi^CD38^hi^ cells from the CD19^+^ B cell population; F: Gating the CD24^hi^CD38^hi^ cells from the CD19^+^ B cell population; G: Gating the CD19^-^CD138^+^ cells in the upper left quadrant, and the CD19^+^CD138^+^ cells in the upper right quadrant from the lymphocyte population.


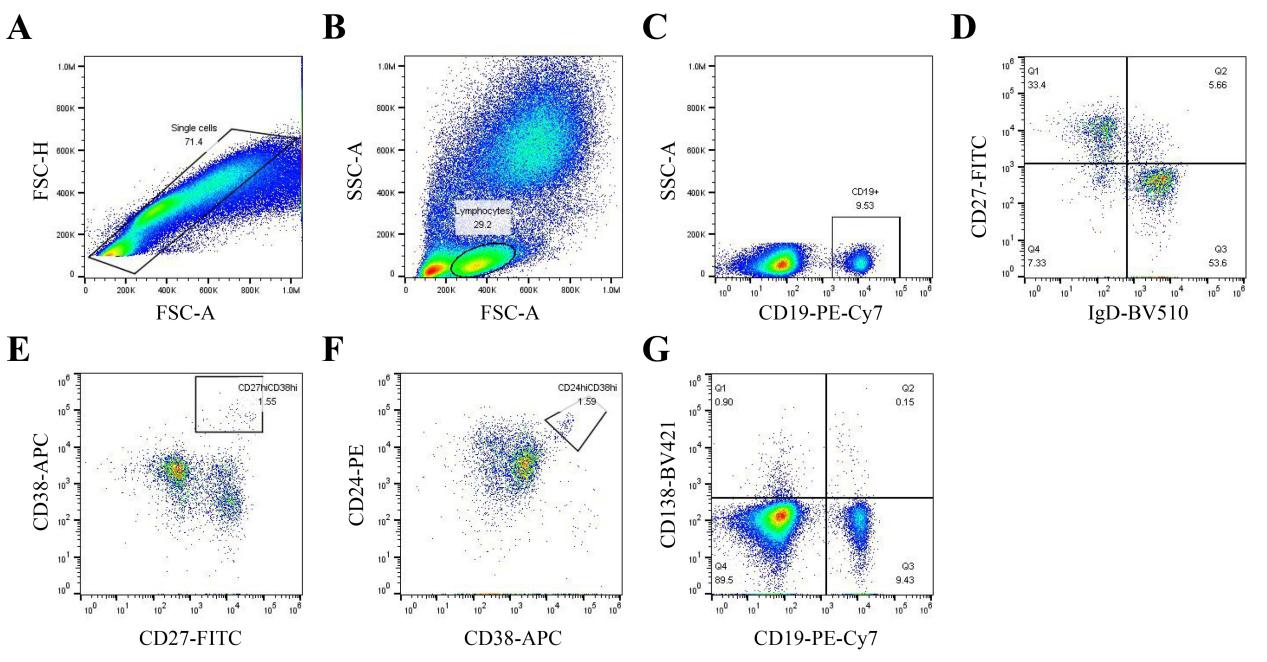


**Supplementary Figure 3. Gating strategy for cell sorting.** A: Gating the single cell population; B: Gating the lymphocyte population; C: Gating the CD4^+^ T cells from the lymphocyte population; D: Gating the CD4^+^CXCR5^+^ Tfh cells from the lymphocyte population; E: Gating the CD19^+^ B cells from the lymphocyte population; F: Gating the CD19^+^CD27^+^ memory B cells and CD19^+^CD27^-^ naive B cells from the lymphocyte population.

**
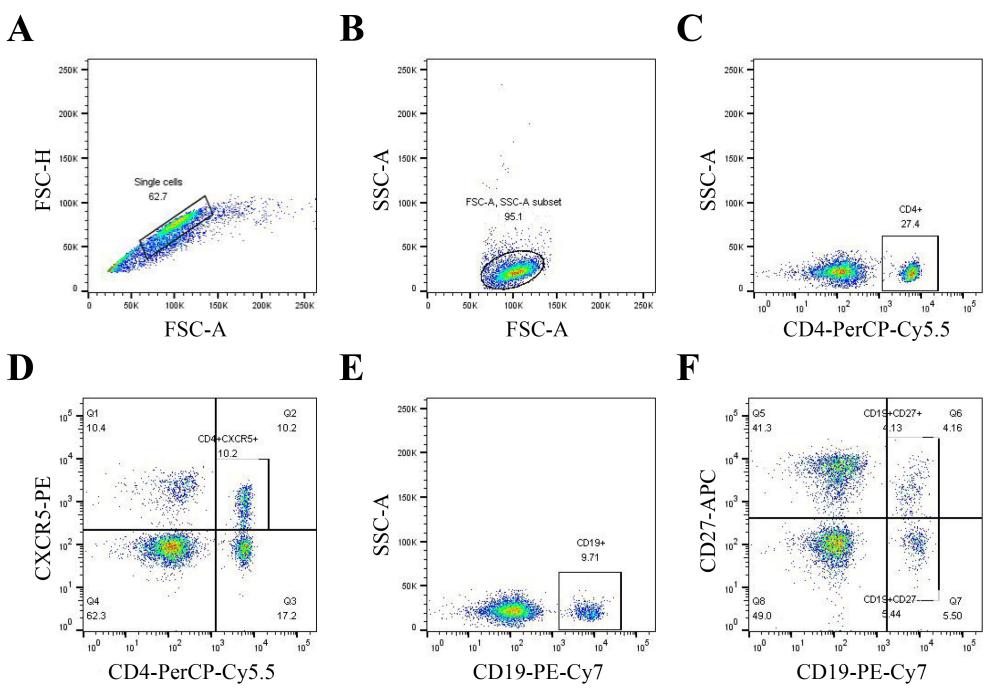
**

**Supplementary Figure 4. Gating strategy for circulating B cell subsets after co-culture of Tfh cells and B cells.** A: Gating the living cells; B. Gating the single cell population from the living cell population; C: Gating the lymphocyte population from the single cell population; D: Gating the CD19^+^ B cells from the lymphocyte population; E: Gating the proliferation cells from the CD19^+^ B cell population; F: Gating the CD27^+^IgD^-^ cells in the upper left quadrant, CD27^-^IgD^-^ cells in the lower left quadrant, CD27^-^IgD^+^ cells in the lower right quadrant, CD27^+^IgD^+^ cells in the upper right quadrant from the CD19^+^ B cell population; G: Gating the CD27^hi^CD38^hi^ cells from the CD19^+^ B cell population.

**
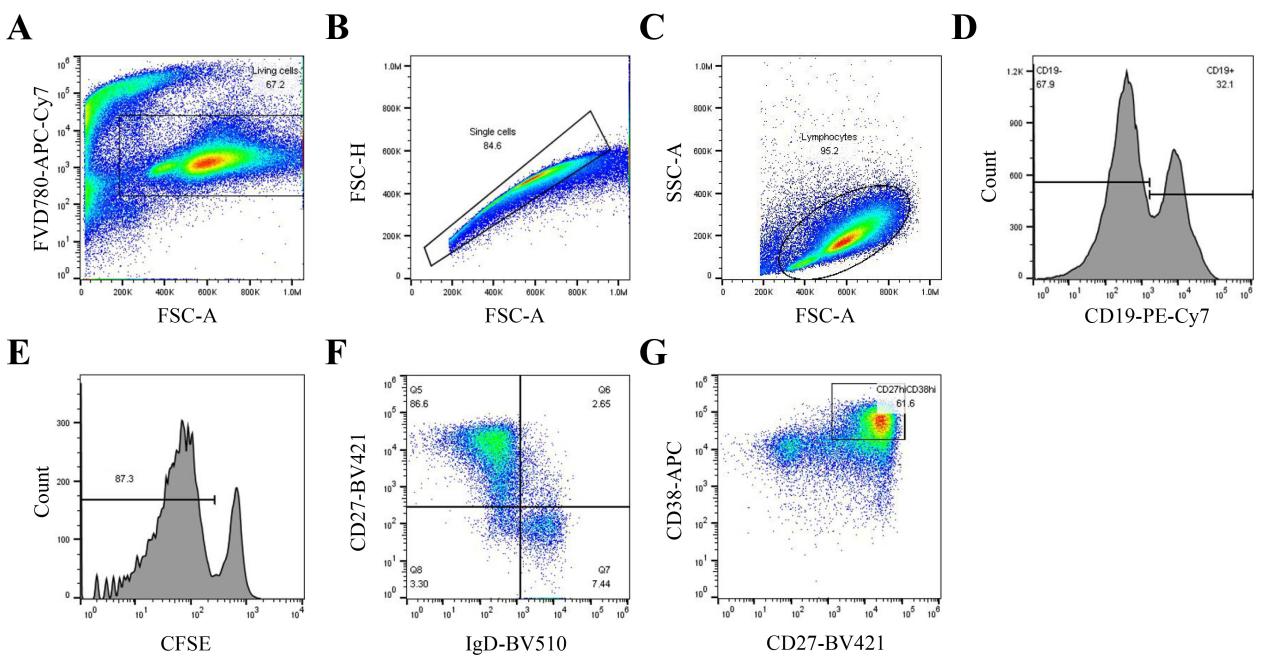
**

**Supplementary Figure 5. Gating strategy for circulating Tfh cell subsets after monoculture of T cells.** A: Gating the living cells; B. Gating the single cell population from the living cell population; C: Gating the lymphocyte population from the single cell population; D: Gating the CD4^+^ T cells from the lymphocyte population; E: Gating the proliferation cells from the CD4^+^ T cell population; F: Gating the CXCR5^+^ cells in the isotype control from the CD4^+^ T cell population; G: Gating the ICOS^+^ cells in the isotype control from the CD4^+^ T cell population; H: Gating the PD1^+^ cells in the isotype control from the CD4^+^ T cell population; I. Gating the BCL6^+^ cells in the isotype control from the CD4^+^ T cell population.

**
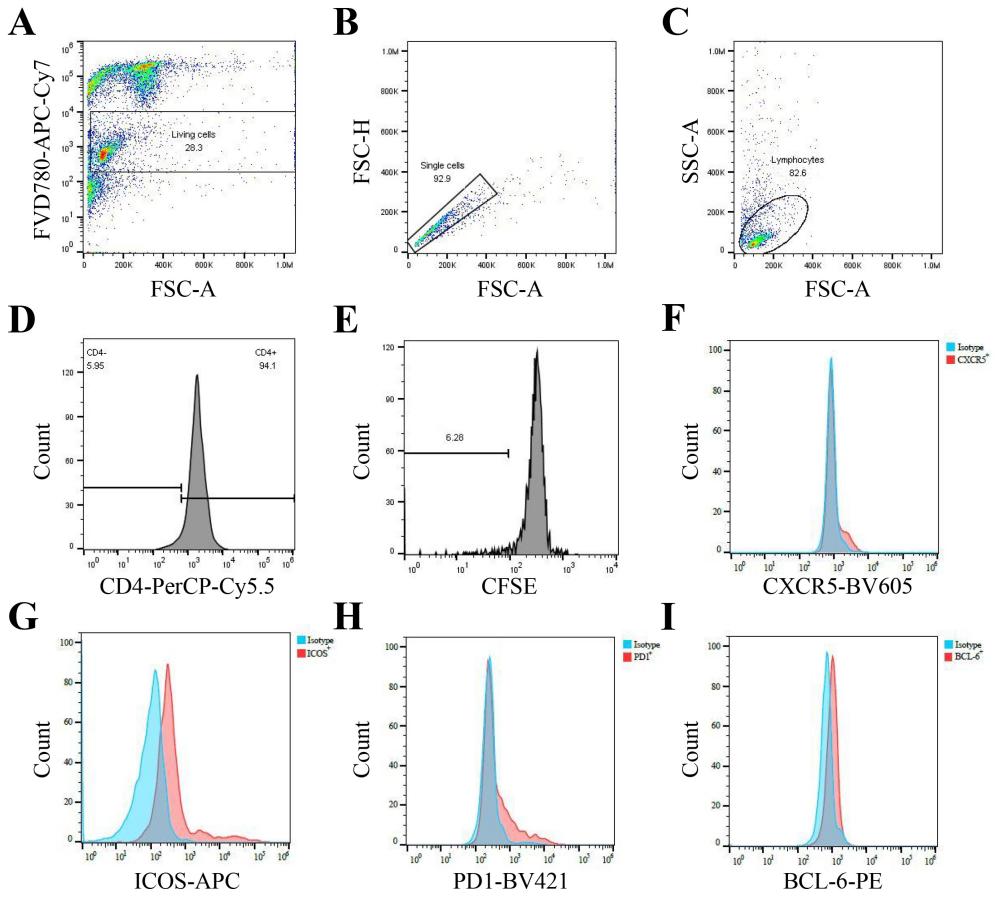
**

**Supplementary Figure 6. Gating strategy for circulating B cell subsets after monoculture of B cells.** A: Gating the living cells; B. Gating the single cell population from the living cell population; C: Gating the lymphocyte population from the single cell population; D: Gating the CD19^+^ B cells from the lymphocyte population; E: Gating the proliferation cells from the CD19^+^ B cell population; F: Gating the CD27^+^IgD^-^ cells in the upper left quadrant, CD27^-^IgD^-^ cells in the lower left quadrant, CD27^-^IgD^+^ cells in the lower right quadrant, CD27^+^IgD^+^ cells in the upper right quadrant from the CD19^+^ B cell population; G: Gating the CD27^hi^CD38^hi^ cells from the CD19^+^ B cell population.

**
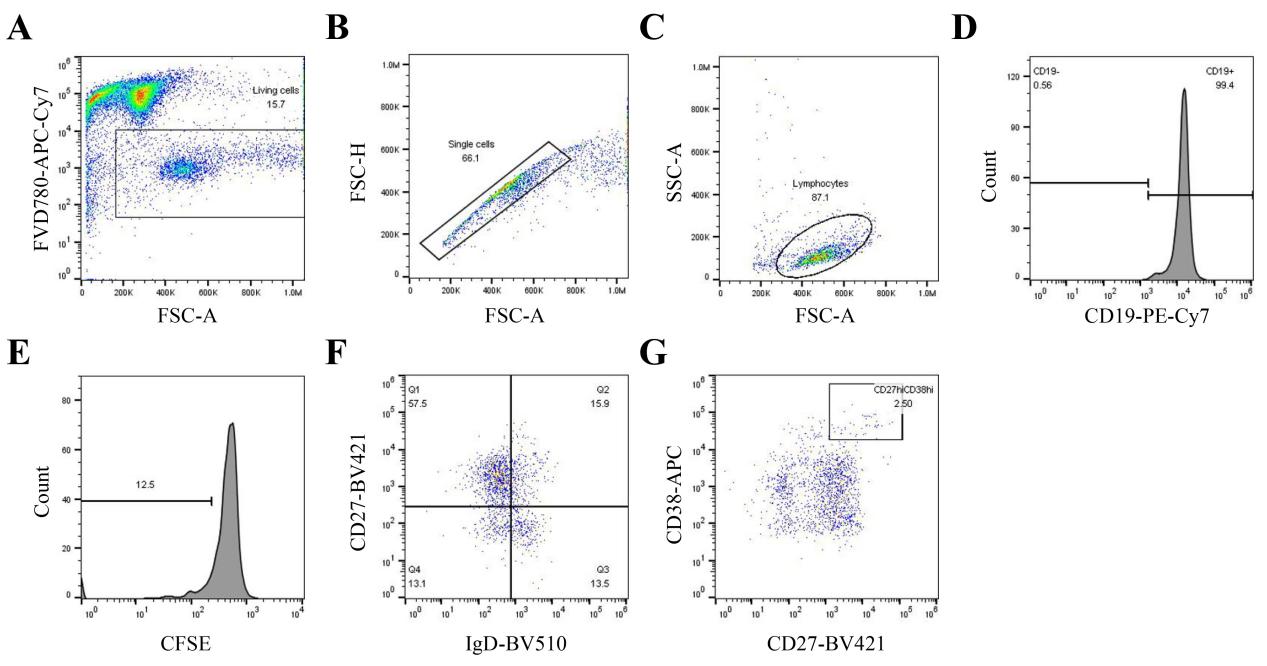
**

**Supplementary Figure 7. Gating strategy for circulating Tfh cell subsets after co-culture of T cells and B cells.** A: Gating the living cells; B. Gating the single cell population from the living cell population; C: Gating the lymphocyte population from the single cell population; D: Gating the CD4^+^ T cells from the lymphocyte population; E: Gating the proliferation cells from the CD4^+^ T cell population; F: Gating the CXCR5^+^ cells in the isotype control from the CD4^+^ T cell population; G: Gating the ICOS^+^ cells in the isotype control from the CD4^+^ T cell population; H: Gating the PD1^+^ cells in the isotype control from the CD4^+^ T cell population; I. Gating the BCL6^+^ cells in the isotype control from the CD4^+^ T cell population.


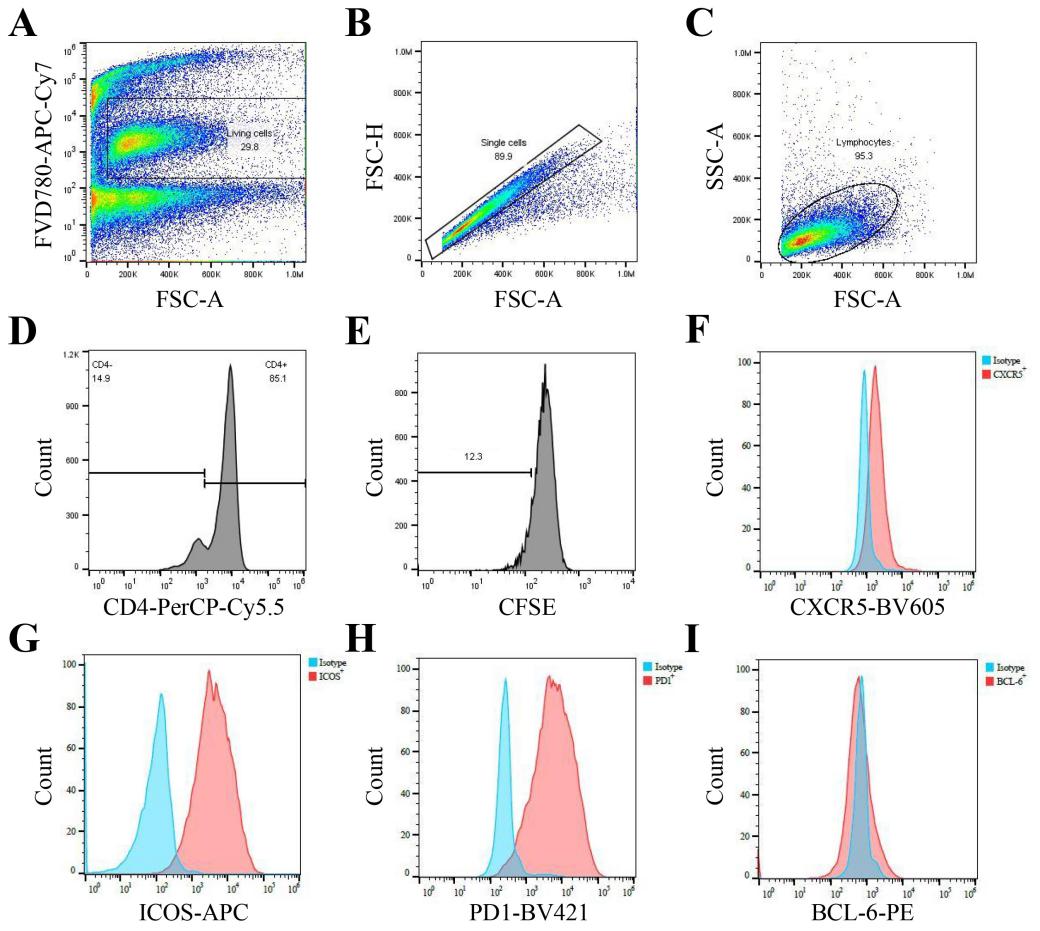


**Supplementary Figure 8. Gating strategy for circulating B cell subsets after co-culture of T cells and B cells.** A: Gating the living cells; B. Gating the single cell population from the living cell population; C: Gating the lymphocyte population from the single cell population; D: Gating the CD19^+^ B cells from the lymphocyte population; E: Gating the proliferation cells from the CD19^+^ B cell population; F: Gating the CD27^+^IgD^-^ cells in the upper left quadrant, CD27^-^IgD^-^ cells in the lower left quadrant, CD27^-^IgD^+^ cells in the lower right quadrant, CD27^+^IgD^+^ cells in the upper right quadrant from the CD19^+^ B cell population; G: Gating the CD27^hi^CD38^hi^ cells from the CD19^+^ B cell population.


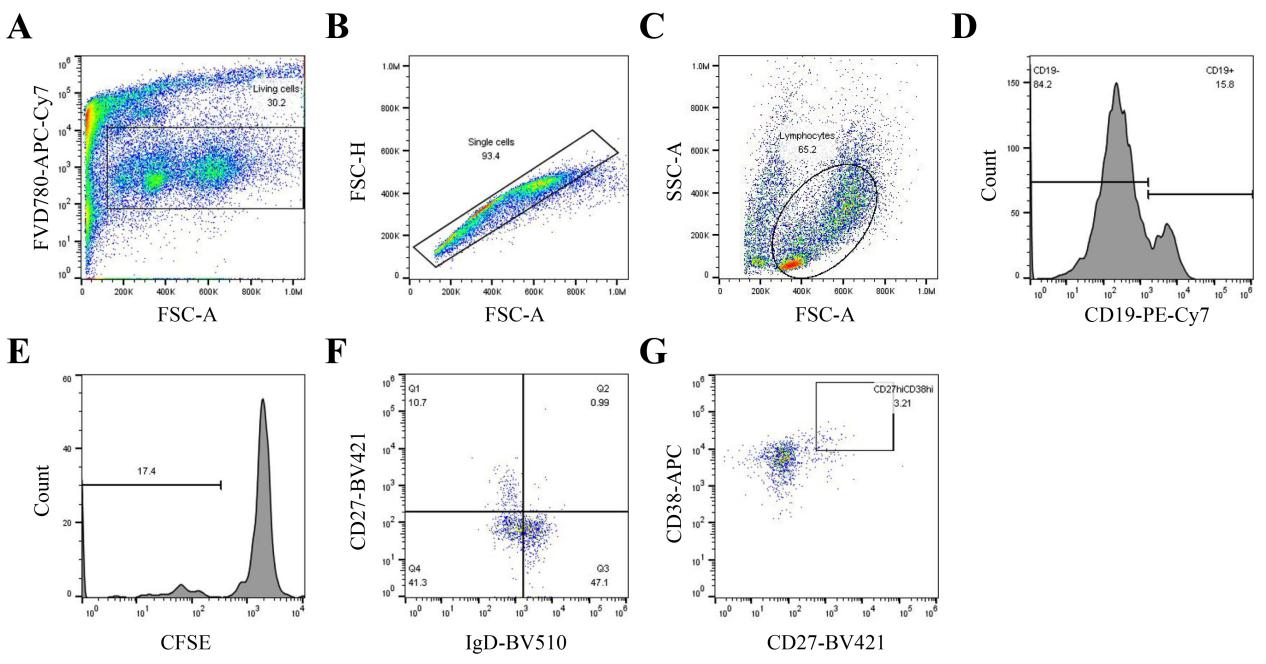


**Supplementary Figure 9. Gating strategy for circulating Tfh cell subsets after co-culture of T cells and B cell subsets.** A: Gating the living cells; B. Gating the single cell population from the living cell population; C: Gating the lymphocyte population from the single cell population; D: Gating the CD4^+^ T cells from the lymphocyte population; E: Gating the proliferation cells from the CD4^+^ T cell population; F: Gating the CXCR5^+^ cells in the isotype control from the CD4^+^ T cell population; G: Gating the ICOS^+^ cells in the isotype control from the CD4^+^ T cell population; H: Gating the PD1^+^ cells in the isotype control from the CD4^+^ T cell population; I. Gating the BCL6^+^ cells in the isotype control from the CD4^+^ T cell population.

**
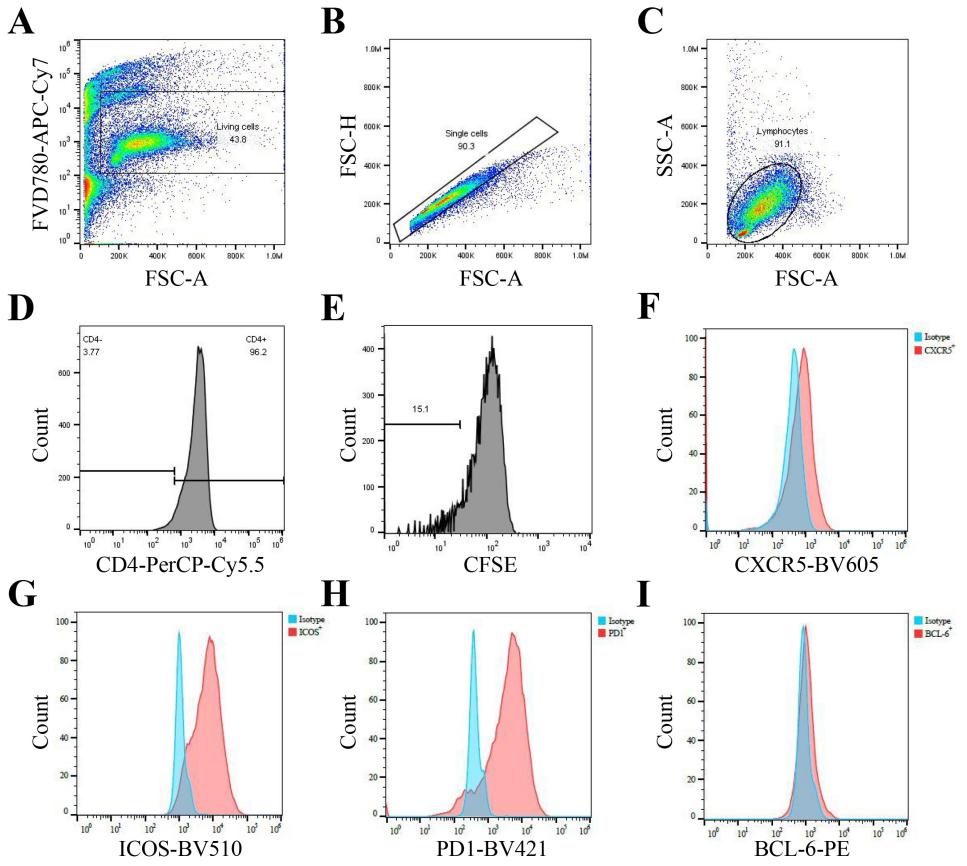
**
